# Determinants of insulin treatment satisfaction among type 2 diabetic older adults

**DOI:** 10.1192/j.eurpsy.2022.1688

**Published:** 2022-09-01

**Authors:** F. Zaouali, N. Lassoued, A. Sondess, W. Alaya, M.H. Sfar

**Affiliations:** University hospital of Mahdia, Endocrinology Department, Mahdia, Tunisia

**Keywords:** Patient Satisfaction, Diabetes Mellitus, Type 2, Insulin, Aged

## Abstract

**Introduction:**

Glycemic control for elderly diabetics is a challenge. Treatment satisfaction reflects this control.

**Objectives:**

To determine the factors associated with insulin treatment satisfaction among type 2 diabetic elderly.

**Methods:**

A cross-sectional study on 86 type 2 diabetic insulin dependent elderly recruited from the outpatient endocrinology consultation during June and July 2021. We applied the Diabetes Treatment Satisfaction Questionnaire (DTSQ) and geriatric assessment scores.

**Results:**

Three quarters of the patients were satisfied with the insulin therapy. Satisfied patients had significantly less history of hospitalization and more regular follow-up. Diabetic neuropathy medications were significantly less taken by satisfied patients. The number of daily insulin injections was significantly higher in the unsatisfied patients. Diabetic foot was significantly more frequent in unsatisfied patients. Satisfied patients were significantly less depressed, more independent in both basic and instrumental activities of daily living, without memory impairment, in better nutritional status and not falling. Higher DTSQ scores were associated with regular follow up (β 7.92, 95% CI 1.83 to 34.3). Lower DTSQ scores were associated with the history of hospitalization (β 0.12, 95% CI 0.02 to 0.58), the taking of medications for diabetic neuropathy (β 0.07, 95% CI 0.09 to 0.51), the high number of insulin injections (β 0.43, 95% CI 0.19 to 0.97) and the presence of diabetic foot (β 0.17, 95% CI 0.01 to 0.38).

**Conclusions:**

Risk factors for patients’ insulin dissatisfaction should be detected early and managed appropriately to improve patients’satisfaction and consequently their well-being.

**Disclosure:**

No significant relationships.

